# Effect of controlled human *Plasmodium falciparum* infection on B cell subsets in individuals with different levels of malaria immunity

**DOI:** 10.1007/s00430-025-00847-x

**Published:** 2025-09-27

**Authors:** Pilar Requena, Gloria Patricia Gómez-Pérez, Matthew B. B. McCall, Diana Barrios, Ruth Aguilar, Julia Fernández-Morata, Marta Vidal, Joseph J. Campo, Carla Sanchez, Maria Yazdabankhsh, B. Kim Lee Sim, Stephen L. Hoffman, Peter Kremsner, Bertrand Lell, Benjamin Mordmüller, Carlota Dobaño, Gemma Moncunill

**Affiliations:** 1 Facultad de Farmacia, Departamento de Medicina Preventiva y Salud Pública, Universidad de Granada, Campus de Cartuja S/N 18170, Granada, Spain; 2 Instituto de Investigación Biosanitaria de Granada (Ibs.GRANADA), Granada, Spain; 3 Centro de Investigación Biomédica en Red de Epidemiología y Salud Pública (CIBERESP), Madrid, Spain; 4 ISGlobal, Barcelona, Catalonia Spain; 5 Facultat de Medicina i Ciències de la Salut, Universitat de Barcelona, Barcelona, Spain; 6 Department of Medical Microbiology and Infectious Diseases, Erasmus MC, Rotterdam, The Netherlands; 7 Department of Medical Microbiology, Radboud Center for Infectious Diseases, Radboud University Medical Center, Nijmegen, The Netherlands; 8 Antigen Discovery, Irvine, CA USA; 9 Leiden University Center for Infectious Diseases (LUCID), Leiden University Medical Center, Leiden, ZA Netherlands; 10 Sanaria Inc., Rockville, MD USA; 11 Centre de Recherches Médicales de Lambaréné (CERMEL), Lambaréné, Gabon; 12 Institute of Tropical Medicine and German Center for Infection Research, University of Tübingen, Tübingen, Germany; 13 Department of Medicine I, Division of Infectious Diseases and Tropical Medicine, Medical University of Vienna, Vienna, Austria; 14 CIBER de Enfermedades Infecciosas (CIBERINFEC), Barcelona, Spain

**Keywords:** *Plasmodium falciparum*, Controlled human malaria infection, B cells, Cytokines

## Abstract

Continuous exposure to *Plasmodium falciparum* (Pf) has been associated with alterations in B cells. We investigated the effect of controlled human malaria infection (CHMI) on B cell phenotypes in individuals with different Pf immunity status: malaria-naïve, immunized with PfSPZ-CVac and semi-immune (lifelong-exposed) volunteers. Compared to naïve, semi-immune but not vaccinated individuals, had increased baseline frequencies of immature B cells (CD19^+^CD10^+^), active naive (IgD^+^CD27^−^CD21^−^) B cells, active atypical (IgD^−^CD27^−^CD21^−^) memory B cells (MBCs), active classical (IgD^−^CD27^+^CD21^−^) MBCs and CD1c^+^-B cells but lower frequencies of some IgG^+^-B cells. The frequencies of CD1c^+^ active atypical MBCs correlated positively with anti-Pf antibodies and negatively with circulating eotaxin levels, while the opposite was observed for IgG^+^ resting atypical MBCs. During early blood-stage infection (day 11 after CHMI), there was an expansion of resting classical (IgD^−^CD27^+^CD21^+^) MBCs in all three groups. Vaccination, compared to placebo, altered the effect of CHMI on B cells, showing a positive association with resting classical MBCs (β = 0.190, 95% CI 0.011–0.368) and active naïve-PD1^+^ (β = 0.637, 95% CI 0.058 to 1.217) frequencies, and a negative one with CD1c^+^ resting atypical MBCs (β = − 0.328, 95% CI − 0.621 to − 0.032). In addition, the sickle cell trait in semi-immune subjects altered the effect of CHMI on several B cells. In conclusion, lifelong but not vaccine exposure to malaria was associated with increased frequencies of multiple B cell subsets, with higher and lower percentages of CD1c and IgG expressing-cells, respectively. A single infection (CHMI) induces changes in B cell frequencies and is modulated by sickle cell trait and malaria-immunity status.

*Clinical Trials Registration* NCT01624961, NCT02115516, and NCT02237586.

**Supplementary Information:**

The online version contains supplementary material available at 10.1007/s00430-025-00847-x.

## Introduction

Malaria is one of the most serious health problems worldwide, with 263 million cases and 597,000 deaths estimated in 2023 [1]. Among *Plasmodium* species causing human malaria*, P. falciparum* (Pf) is the major responsible for the high rates of mortality. Different efforts are ongoing to fight malaria disease, and one of the strategies is vaccination. Two malaria vaccines, RTS,S/AS01_E_ (Mosquirix®) and R21/Matrix-M™, are recommended by the World Health Organization for widespread use among children living in regions with moderate to high Pf malaria transmission [1]. However, since duration and level of protection are not optimal [2, 3], next-generation vaccines are being developed. One candidate with promising results is the Sanaria PfSPZ-CVac vaccine, which consists of intravenous (IV) injection of Pf sporozoites (PfSPZs) together with chloroquine as a chemoattenuating drug. In a clinical trial conducted in Tübingen (Germany), PfSPZ-CVac immunization resulted in full protection against controlled human malaria infection (CHMI) 8–10 weeks after the last vaccine dose [4], a finding that could be reproduced in subsequent trials [5, 6]. Despite the logistic challenges of large-scale deployment, due to its high efficacy, PfSPZ-CVac is an excellent model/proof of concept for second generation live attenuated PfSPZ vaccines such as those based on genetically attenuated parasites [7].

CHMI, consisting of injecting drug-sensitive Pf and closely monitoring parasitemia to administer antimalarial drugs at the first sign of infection, is a powerful tool for evaluation of the efficacy of novel malaria vaccines and drugs (reviewed in [8]). CHMI provides also a unique opportunity to study in depth the early immune response to Pf infection.

Immunity to clinical malaria is slowly acquired over time through repeated natural infections; however, sterile immunity to Pf infection is rarely developed. As a result, in highly endemic areas adults frequently have asymptomatic and submicroscopic infections, i.e. low parasitemias only detected by qPCR [9]. Moreover, naturally acquired immunity to clinical malaria may diminish when frequent exposure to *Plasmodium spp.* is discontinued, with several studies reporting declines of Pf-specific antibodies within months and even weeks after an initial potent response [10–13] in contrast to the antibody responses against many viruses and non-replicating antigens used in vaccines that last decades [10, 14]. In this regard, we and others have demonstrated clear associations between high levels of exposure to *Plasmodium spp.* and alterations in peripheral B lymphocyte populations that are responsible for antibody production. An expansion of CD10^+^ immature B cells has been seen in children with acute episodes of Pf malaria, which reached up to 25% of the CD19^+^ B lymphocyte subset [15]. The expansion of immature B cells has also been described in HIV [16] and other human immunodeficiency states [17] implying they are associated with chronic immune stimulation. In addition, a subset of CD21^−^CD27^−^ memory B cells (MBCs), termed as “exhausted” or “atypical” MBC are expanded in malaria, HIV and other infections, and correlated with anti-*Plasmodium* antibody levels [18–24]. It is not clear whether these malaria-related atypical MBCs are dysfunctional compared to classical MBCs. On the one hand, atypical MBCs have a transcriptional profile distinguishable from the classical ones [25], with a reduced expression of genes involved in BCR signaling [26] and antigen recognition [27], and lower surface IgG levels [24, 26, 28]. On the other hand, signs of proliferative activity and secretion of antibodies have been described in atypical MBCs [28]. It also remains uncertain if a single or only repeated exposure to malaria parasites is sufficient to induce these alterations in B cell subsets. Most studies have been performed in malaria endemic regions, but one study reported an expansion of atypical MBCs in malaria-naïve Dutch individuals after a single CHMI [29]. In addition, the levels of chemokine CCL11 (eotaxin) have been negatively correlated with atypical MBC [24], as well as *P. vivax* infection and Pf parasitemia [30, 31]. Furthermore, frequencies of atypical MBCs had a positive correlation with serum concentrations of the proinflammatory cytokines tumor necrosis factor (TNF-α) and interleukin (IL)-8 in a cohort with a lifelong exposure to malaria [24].

The objectives of this work were (1) to compare the B cell profiles of individuals with different Pf immunity status: malaria-naïve, PfSPZ-CVac-immunized and malaria semi-immune individuals with lifelong exposure to Pf in Africa and (2) to evaluate the effect of a single Pf infection via CHMI on these groups.

## Materials and methods

### Study participants

**Fig. 1:**
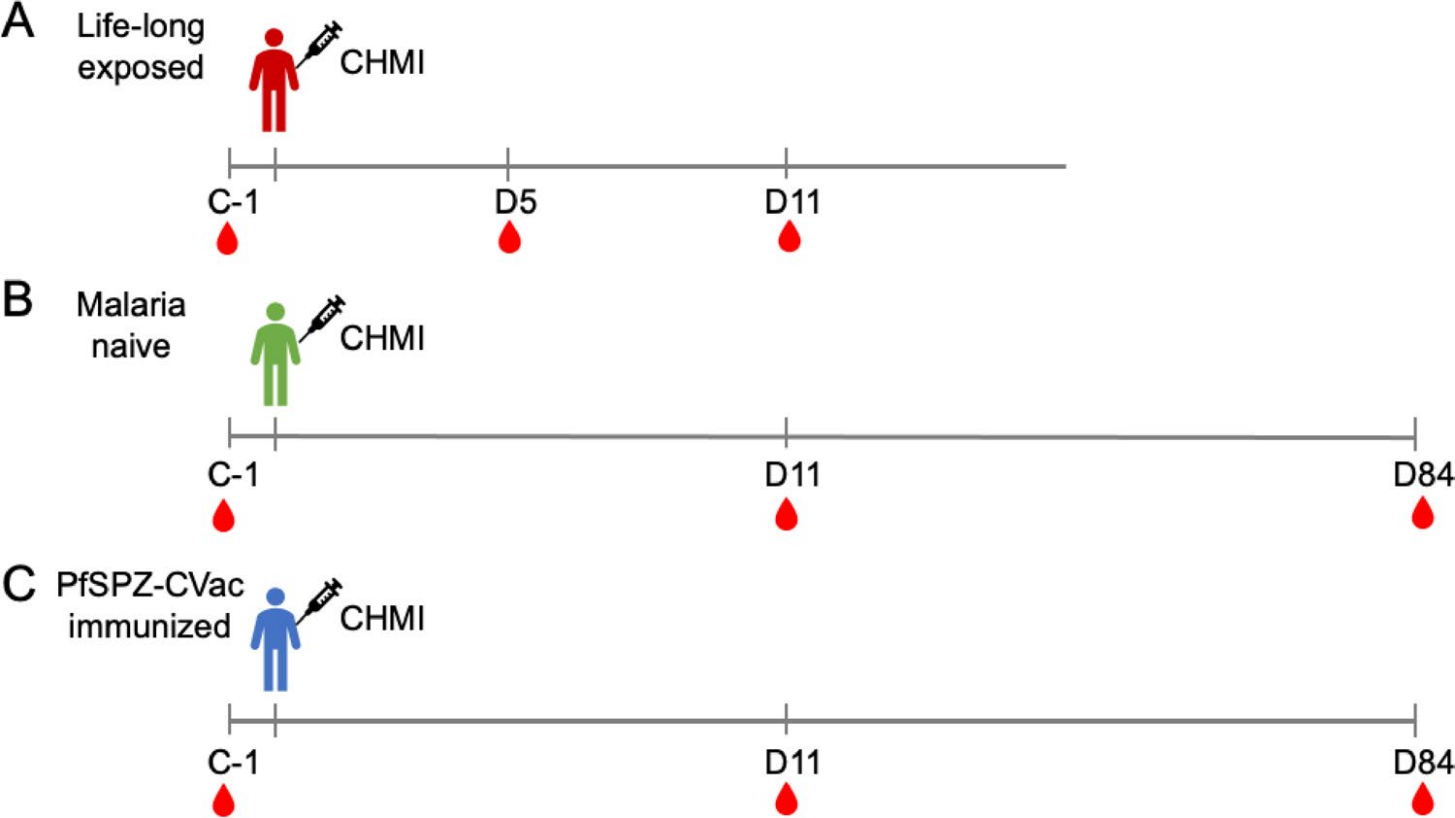
Scheme of clinical trials. **A** Life-long exposed individuals trial (LACHMI-001). **B** Malaria naïve individuals clinical trial (TÜCHMI-001). **C** Malaria naïve PfSPZ-CVac vaccinated individuals trial (TÜCHMI-002). Days of blood sample collection for PBMC cryopreservation and subsequent B cell analysis are indicated

This study was performed in the context of three CHMI trials (Fig. 1) that differed in the level of malaria exposure of the participants: malaria lifelong exposed individuals (LACHMI-001 trial), malaria naïve individuals (TÜCHMI-001 trial) and malaria naïve PfSPZ-CVac vaccinated individuals (TÜCHMI-002 trial). The protocols have been published previously [4, 32, 33].

The LACHMI-001 clinical trial (NCT02237586) [32] was designed to study the effect of lifelong malaria exposure as well as sickle cell trait on healthy adults aged 18‒30 years following experimental infection with 3,200 PfSPZs (strain NF54) administered by direct venous inoculation (DVI) at CERMEL (Gabon) from July 2014 to February 2016. Volunteers with no history of antimalarial drug intake were allocated into three groups: non-immunes (N = 5) minimally exposed to malaria (Europeans living temporally in Gabon), and semi-immunes with a lifelong malaria exposure (Gabonese individuals from Lambaréné), further segregated depending on the presence (hemoglobin [Hb] AS, N = 9) or absence (HbAA, N = 11) of sickle cell trait. Peripheral blood was collected one day before (C-1) and five (D5) and 11 (D11) days after the CHMI, and peripheral blood mononuclear cells (PBMCs) were cryopreserved. Non-immune participants were treated when parasitemia was detected, and semi-immunes when parasitemia and clinical symptoms were detected or at D28 if they were not treated before [32]. In this study eight semi-immune subjects with sufficient PBMCs were analyzed (HbAS, n = 5; HbAA, n = 3).

The TÜCHMI-001 clinical trial (NCT01624961) [33] aimed to determine the best dose of PfSPZs administered by IV injection through an indwelling catheter for a CHMI regimen. The study was performed at the Eberhard Karls University of Tübingen (Germany) from June to December 2012, with a verification group with the highest dose of parasites administrated by DVI at the Hospital Clínic (Barcelona, Spain) from December 2012 to July 2013 (NCT01771848) [34]. Participants were healthy individuals (N = 36) age 18–45 years with no history of malaria. Dose-escalation of PfSPZ (NF54) started with 50 and ended with a maximal dose of 3,200 PfSPZ. Peripheral blood was collected one day before the PfSPZ inoculation (C-1), 11 (D11), and 84 (D84) days after, and PBMCs were cryopreserved [33]. The number of individuals analyzed here was seven, all from the maximal dose group (3,200 PfSPZ) from TÜCHMI-001 clinical trial.

The TÜCHMI-002 clinical trial (NCT02115516) aimed at establishing a safe and well-tolerated vaccine regimen using three different doses by DVI (3.2 × 10^3^, 1.28 × 10^4^ and 5.12 × 10^4^) of purified, cryopreserved PfSPZs administered to malaria-naïve healthy adult volunteers taking chloroquine (PfSPZ-CVac vaccine). To assess vaccine efficacy, placebo and PfSPZ-CVac immunized individuals underwent CHMI 8–10 weeks later with 3,200 PfSPZ (NF54) by DVI. The trial was carried out in Tübingen (Germany) from May to July of 2014. Peripheral blood was collected at C-1, D11, D84, and PBMCs were cryopreserved [4]. The number of subjects analyzed here was 33 (placebo, n = 12; PfSPZ-CVac vaccinated, n = 21).

### Ethical approval

For all studies, written informed consent was obtained from all participants. The TÜCHMI-001 (699/2011BO1) and TÜCHMI-002 (537/2013AMG1) studies were approved by the ethics committee of the University Clinic and the Medical Faculty of the University of Tübingen, and the Hospital Clinic and the Hospital de la Santa Creu i Sant Pau ethics committees in Barcelona. The LACHMI-001 study received approval by the Gabonese National Ethics Committee (Comité National d’Ethique de la Recherche, 0005/2014/CNE/SG/PR) and was conducted under the USA FDA Investigational New Drug application. All these studies strictly followed the principles of the Declaration of Helsinki in its sixth revision as well as the Good Clinical Practice guidelines.

### Detection of Pf

Pf infection was diagnosed by thick blood smear (TBS) microscopy and by 18S qPCR. From day 5 until antimalarial treatment, blood samples were withdrawn daily. Quantitative TBS were prepared as described elsewhere [35] and qPCR was performed after all the samples were collected, using published protocols [4, 32, 33].

### Isolation of plasma and PBMCs

Plasma was separated from the cellular fraction of heparinized peripheral whole blood by centrifugation at 600 g for 10 min at room temperature within 2‒4 h of collection, aliquoted and stored at − 80 °C. A density gradient medium (Lymphoprep 4X250mL, PALEX, Cat 1114545) was used to obtain PBMCs that were frozen in fetal bovine serum with 10% of dimethyl sulfoxide and stored in liquid nitrogen until analysis.

### Immunophenotyping and gating strategy

PBMC samples were thawed and processed at ISGlobal (Spain) in three periods of time for logistic reasons: (i) all LACHMI-001 and 11 TÜCHMI-002 samples, (ii) 2 TÜCHMI-001 and 7 TÜCHMI-002 samples and (iii) 5 TÜCHMI-001 and 15 TÜCHMI-002 samples. Samples were thawed by adding RPMI 1640 medium supplemented with 10% fetal bovine serum (Life Technologies) and 0.5µL/mL benzonase (Novagen-Merck, Cat 70664-3), and were centrifuged 500xg for 7 min at RT. After thawing, PBMC viability was measured on a Guava® Cytometer (PC550IG-C4C/0746-2747) using ViaCount Reagent (Merck-Millipore, Cat 4000-0041). Samples with viabilitites below 70% were excluded.

**Fig. 2:**
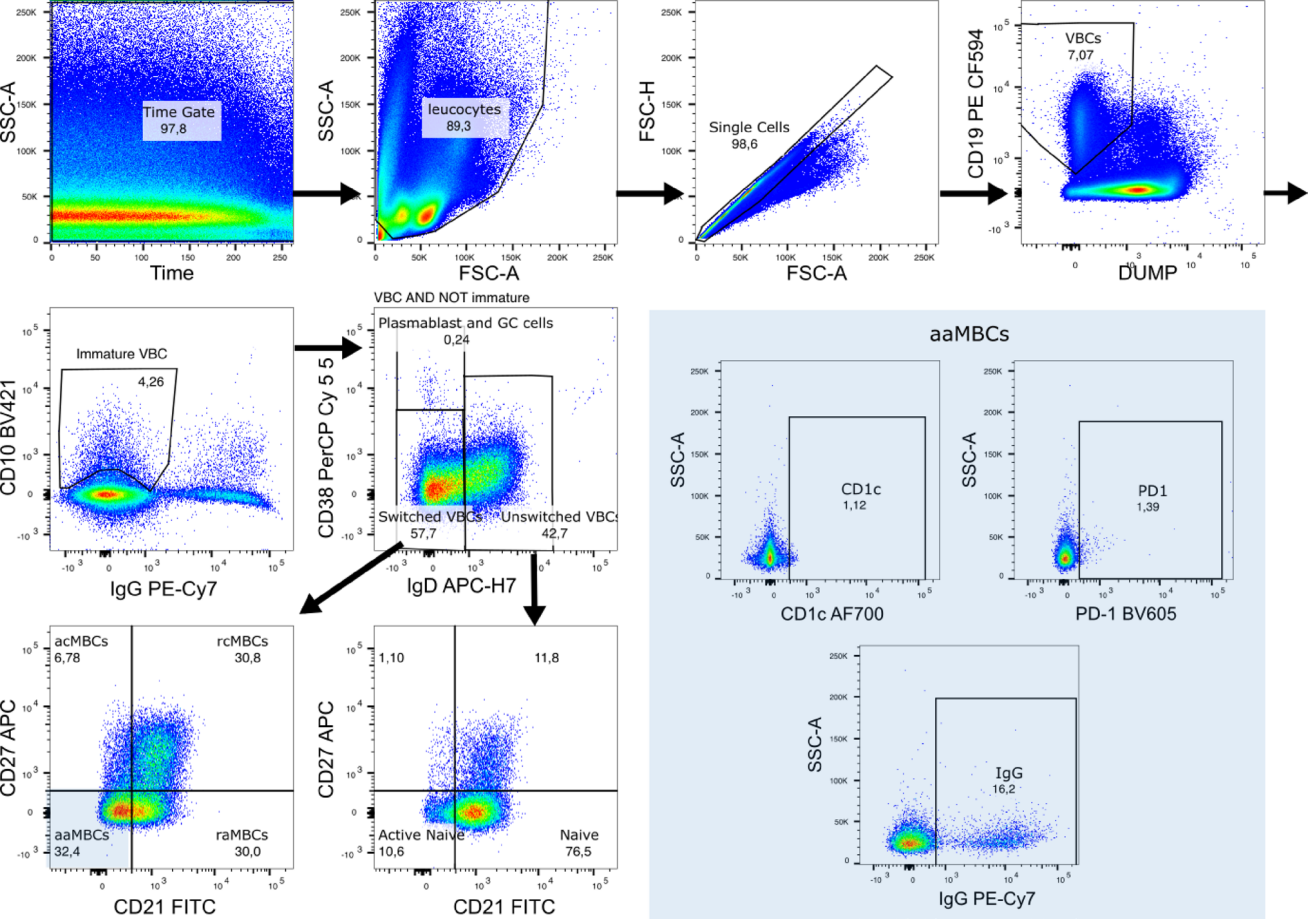
Gating strategy. Viable B cells (VBC) were gated based on CD19 ^+^ and DUMP^-^ (DUMP channel included life/dead staining, CD3, CD14, and CD16); immature B cells were CD10 ^+^ and IgG- VBC; plasmablasts and germinal center cells were gated within the in VBC AND NOT immature cells (IgD^−^ and CD38^++^); switched VBC were gated within VBC AND NOT immature VBCs and were CD38^+^ (not high) and IgD^−^; acMBCs abbreviates active classical memory B cells (MBCs) (Switched: CD21^−^ CD27^+^); rcMBCs, resting classical MBCs (switched: CD21^+^CD27^+^); aaMBC, active atypical MBCs (switched: CD21^−^CD27^−^); raMBCs, resting atypical MBCs (switched: CD21^+^CD27^−^); unswitched (VBC not immature VBCs, not CD38^++^ and IgD^+^); resting naïve (unswitched: CD21^+^CD27^−^); active naïve (unswitched: CD21^−^CD27^−^)

Between 5 × 10^5^ and 1 × 10^6^ PBMCs per sample were used for B cell staining. Cell suspensions were stained with LIVE/DEAD® Fixable Aqua Dead Cell Stain Kit (Invitrogen, Cat L34957), washed and blocked with bovine serum albumin 0.5% for 15 min. After washing, cells were stained with an 11-color panel. Supplementary Table 1 summarizes the antibodies used. For compensation control, BD Comp Beads (BD, Cat 552,843) were used. To establish the gates for positive events, Fluorescence Minus One (FMO) controls were performed which consisted on staining samples with all the fluorophores used in the panel except one of them. The gating strategy is summarized in Fig. 2.

First, leukocytes were gated using a time event gate and selecting singlets. Viable B cells (VBCs) were gated by CD19-expression and by excluding CD3^+^, CD14^+^, CD16^+^, dead and apoptotic cells. Immature VBCs were gated as CD10^+^ and IgG^-^ within CD19^+^ live cells, whereas mature VBCs were CD10^−^. Mature VBC were divided into switched (IgD^−^), unswitched (IgD^+^ CD38^−/low^), plasmablast cells and germinal center cells (PCGCs) (IgD^−^ CD38^++^) populations. Of note, PCGCs can also include pre-germinal cells and recent germinal cells. Switched and unswitched populations were further segregated by their expression of CD21 and CD27. Within the switched population, MBCs were classified as active classical (acMBCs) (CD27^+^ CD21^−^), resting classical (rcMBCs) (CD27^+^ CD21^+^), active atypical (aaMBCs) (CD27^−^ CD21^−^) and resting atypical (raMBCs) (CD27^−^ CD21^+^). The unswitched (IgD^+^) population was classified as resting naïve (CD27^−^ CD21^+^) and active naïve (CD27^−^ CD21^−^) B cells. B cell subpopulations are reported here as percentage of total VBCs. CD1c^+^, IgG^+^ and PD1^+^ cells were gated within each B cell subset. We included CD1c and PD1 as activation and immunoregulatory markers [36–38]. Cell acquisition was performed on a BD LSR II Fortessa cytometer and the data analysis was performed on FlowJo software version v10.

### Antibody and cytokine analyses

Plasma samples were collected 1–2 days before CHMI (C-1), and on the following days after: D7, D11-13, D19 (only in the LACHMI-001 cohort), D28 (only in the TÜCHMI-001 and LACHMI-001 cohorts), and D84 (only for TÜCHMI-001 and TÜCHMI-002). Anti-IgG to 21 Pf antigens were measured by quantitative suspension array technology using the xMAP™ platform (Luminex Corp., Austin, Texas) and their levels were expressed as median fluorescence intensity, as described [39, 40] & [Gómez-Pérez et al., [41]]. Plasma cytokines were measured by means of the xMAP™ technology (Luminex Corp., Austin, Texas) at time points C-1, D7, D13, D19 and D28 using the Cytokine Human Magnetic 30-Plex Panel from Life Technologies™ as described before [31]. The kit included the following proteins representing major cytokine families (Th1, Th2, Th17), chemokines (proinflammatory and regulatory) and growth factors: epidermal growth factor (EGF), fibroblast growth factor (FGF), granulocyte colony-stimulating factor (GCSF), granulocyte–macrophage colony-stimulating factor (GMCSF), hepatocyte growth factor (HGF), vascular endothelial growth factor (VEGF), TNF-α, interferon (IFN)-α, IFN-γ, IL-1 receptor agonist (RA), IL-1β, IL-2, IL-2R, IL-4, IL-5, IL-6, IL-7, IL-8, IL-10, IL-12(p40/p70), IL-13, IL-15, IL-17, IFN-γ induced protein (IP-10), monocyte chemoattractant protein (MCP-1), monokine induced by IFN-γ (MIG), macrophage inflammatory protein (MIP)-1α, MIP-1β and regulated on activation normal T cell expressed and secreted (RANTES) and CCL11 (eotaxin). Concentrations in pg/mL were log_10_ transformed for analysis.

### Statistical analysis

Cell population frequencies were calculated with respect to the VBCs, therefore, alterations in one population were detected as changes on the other cell subsets. To improve statistical power, all malaria-naïve individuals (TÜCHMI-001 and placebo TÜCHMI-002) were grouped and termed “malaria naïve”.

The distributions of quantitative variables were analyzed using the skewness and kurtosis tests for normality. Most frequencies of B cells did not follow a Gaussian distribution (data not shown) and therefore non-parametric statistical tests were chosen for the analysis. Baseline frequencies of B cell subsets were compared between the three exposure groups (naïve, vaccinated and semi-immune) by Kruskal–Wallis followed by Dunn’s test, corrected with the Bonferroni method for multiple comparisons. The effect of the CHMI was evaluated independently in each exposure group by means of the Friedman’s test, followed by two-by-two comparisons with Bonferroni’s correction. To assess whether previous vaccination affected the B cell changes induced by CHMI, we performed generalized estimated equations and explored the interaction between time and group allocation (placebo vs. vaccine) in the TÜCHMI-2 cohort. The same analysis was performed to evaluate the interaction between time and sickle cell trait (HbAA vs HbAS) in the LACHMI-001 cohort. To analyze the association between the B cell frequencies and other population variables and infection after CHMI, individual or multivariable logistic regressions were estimated with the samples from LACHMI-001 and the PfSPZ-CVac-vaccinated group, estimating the odds ratios (OR) and 95% confidence intervals (CI).

The correlations between B cell frequencies and antibody levels or cytokine concentrations were assessed by Spearman’s test at baseline with the naïve, semi-immune and vaccinated donors data together and separately.

Statistical significance was defined at *p* < 0.05, and at *p* < 0.1 a trend was considered. All the statistical analyses and graphs were performed using Stata v17 (Stata Corp., College Station, TX, USA, 2017), SPSS version 28.0.1.0 (IBM Corp., Armonk, N.Y., USA) and R (corrplot and tidyverse packages) [42, 43]. Figures 6 and 7 were elaborated using chatgpt.

## Results

### Population

The clinical characteristics of study participants are summarized in Table 1. There were more males than females and the median body mass index was in the normal range. After CHMI, all the individuals in the TÜCHMI-001 and placebo-TÜCHMI-002 groups became parasitaemic, while only 29% of the vaccinated-TÜCHMI-002 did. In the LACHMI-001 cohort (semi-immune), 75% of the individuals had a positive smear and 88% were positive by qPCR. In addition, all the individuals with HbAA developed microscopic parasitaemia, while among the participants with HbAS, 40% and 80% got positive smears and qPCR, respectively.

**Table 1:** Population characteristics

	LACHMI-001	TÜCHMI-001	TÜCHMI-002 placebo	TÜCHMI-002 vaccinated	Naive (TÜCHMI-001 + placebo)	Total
N	8	7	12	21	19	48
Sex (F:M)	2:6	1:6	8:4	7:14	9:10	18:30
Age mean (SD)	21.9 (2.30)	27.8 (2.27)	25.43 (4.12)	27.14 (4.86)	26.30 (3.58)	
BMI mean (SD)	21.84 (1.82)	23.89 (3.08)	21.66 (3.36)	24.99 (3.09)	22.48 (3.36)	23.47 (3.28)
Hb status (HbAA: HbAS)	3:5	N/A	N/A	N/A	N/A	N/A
Microscopic malaria TBS test (pos:neg)	6:2	7:0	12:0	6:15	19:0	31:17
Submicroscopic malaria qPCR test (pos:neg)	7:1	7:0	8:0	6:15	15:0	28:16
Days to infection geometric mean (SD)	20.3 (4.4)	11.0 (0.4)	12.1 (2.3)	12.3 (1.1)	11.69 (1.87)	13.5 (4.1)

LACHMI-001: semi-immune Gabonese individuals with lifelong malaria exposure that donate blood samples one day before, and five and 11 days after the CHMI. TÜCHMI-001: healthy malaria-naive German individuals, with blood samples extracted one day before the CHMI, 11 and 84 days after. TÜCHMI-002: clinical trial performed in malaria-naive German individuals aimed at establishing a safe and well-tolerated PfSPZ-CVac vaccine; placebo and vaccinated individuals underwent CHMI 8–10 weeks later. Peripheral blood was collected one day before the CHMI, 11 and 84 days after. For some of the analyses, all naive individuals (TUCHMI-001 and TUCHMI-002 -placebo) were grouped. BMI: body mass index. Hb: hemoglobin. TBS: thick blood smear.

### Baseline B cell frequency differences between exposure groups

**Fig. 3:**
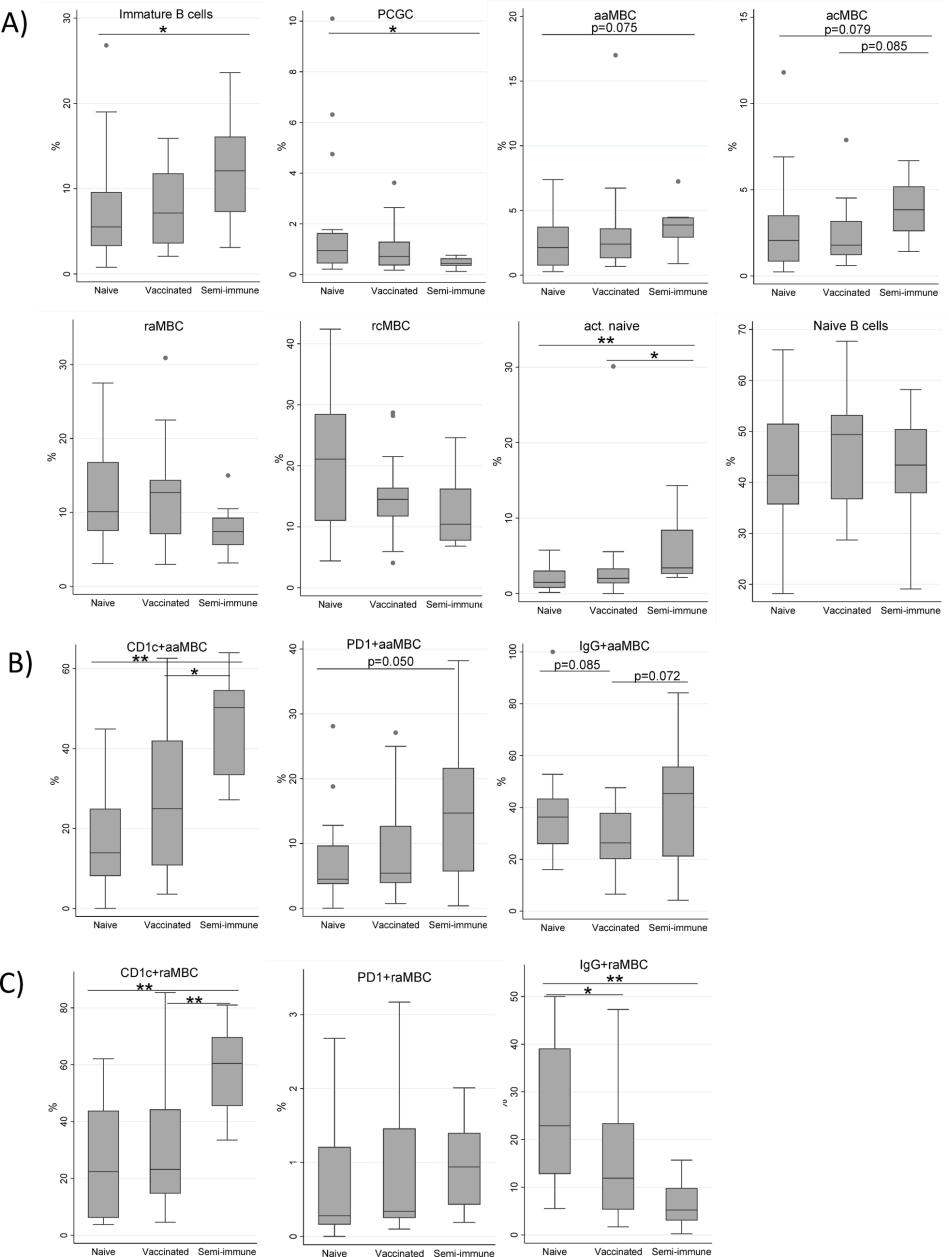
Differences in frequencies of B cells between groups at baseline. Boxplots show, for the different study populations (naïve (n = 19), vaccinated (n = 21) and semi-immune individuals (n = 8)), **A** the baseline percentages of B cell populations, **B** the frequencies of active atypical memory B cells (aaMBCs) expressing the different markers, **C** the frequencies of resting atypical memory B cells (raMBCs) expressing the different markers. Outliers in the plot of PD1 + raMBC are excluded. The values for the remaining B cell populations expressing CD1c, PD1 and IgG are provided in the Supplementary Table 2. Median, and 25th and 75th percentiles (lower and upper hinge respectively) are represented as boxes. *p < 0.05, **p < 0.01, other *p*-values < 0.1 are reported with numbers. P-values correspond to Dunn’s test corrected for multiple comparisons with the Bonferroni method

Compared to naïve, semi-immune participants had higher percentages of immature B cells, aaMBC, acMBC and active naïve B cells, and lower percentages of PCGC at baseline (Supplementary Table 2 and Fig. 3A). However, some of these differences were not statistically significant after adjusting for multiple testing. In addition, for all B cell subsets, semi-immune donors had higher frequencies of CD1c-expressing cells than naïve individuals (Supplementary Table 2 and Fig. 3B, C). Similar results were observed when semi-immune were compared to vaccinated individuals. In contrast to CD1c-expressing cells, semi-immune individuals had lower percentages of IgG^+^ cells for many B cell subsets compared to naïve and vaccinated donors, except aaMBCs, for which they had a higher percentage (Supplementary Table 2 and Fig. 3B, C). With regards PD1-expressing cells, only aaMBCs showed different frequencies among exposure groups, with semi-immune donors having more PD1^+^ aaMBC cells than naïve individuals. No differences in the frequencies of B cell subsets were observed between naïve and vaccinated individuals, with the exception of a trend towards lower IgG^+^ aMBC (both resting and active) in vaccinated than naïve individuals (Supplementary Table 2 and Fig. 3B, C).

In summary, cumulative but not a single (vaccine) malaria exposure was associated with increased frequencies of many B cell subsets, with higher and lower percentages of CD1c and IgG expressing-cells, respectively. The aaMBC subset presented a different behavior, as semi-immune donors had more IgG^+^ aaMBC and PD1^+^aaMBC cells than vaccinated and naïve individuals, respectively.

### Effect of CHMI on each malaria-exposure group and B cell subset

**Fig. 4:**
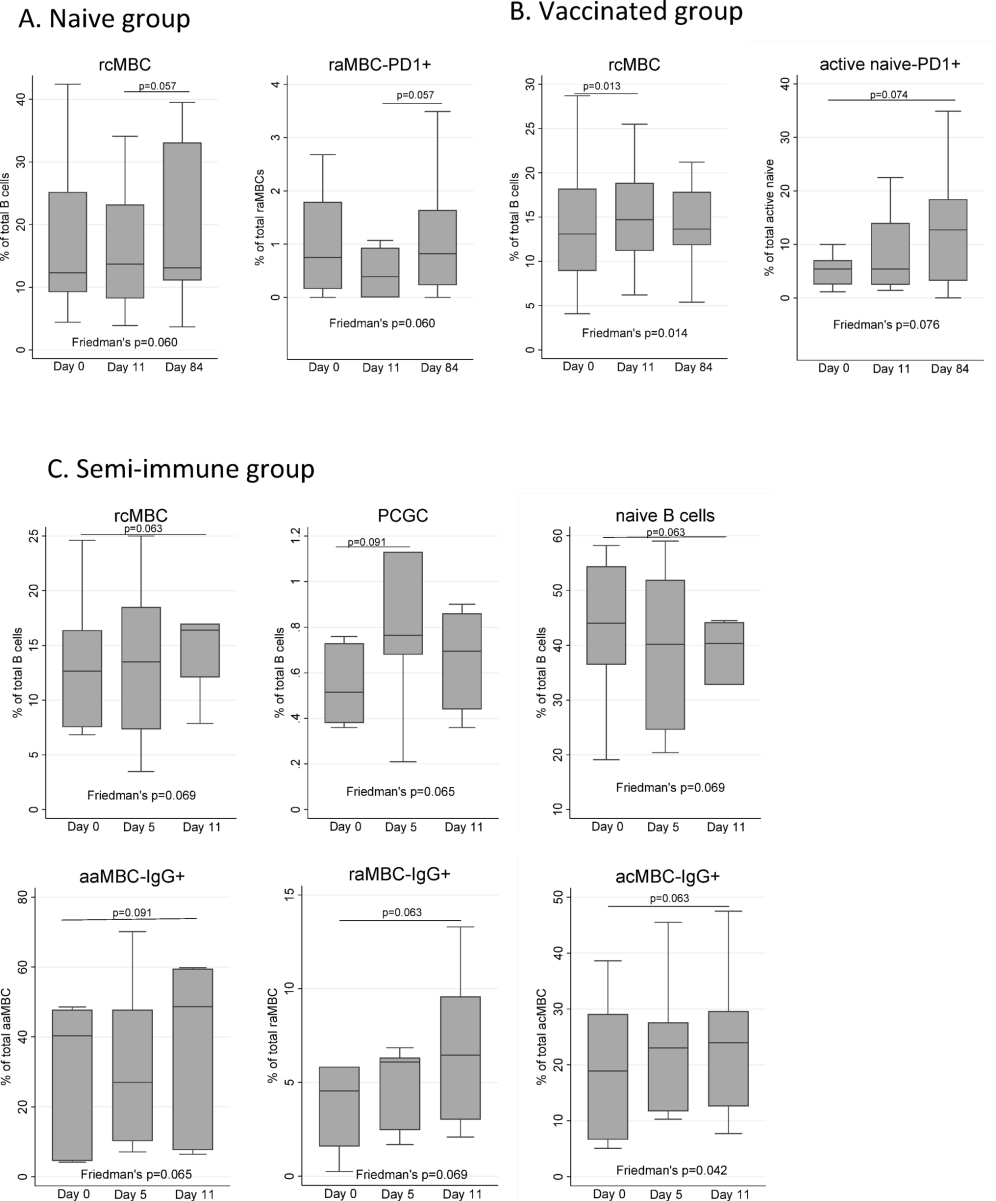
Effect of CHMI on B cell subsets distribution. Boxplots show the percentages of selected B cell populations before (Day 0) and at different timepoints after the CHMI, in the categorized groups: **A** naive, **B** vaccinated, and **C** semi-immune individuals. Median, and 25th and 75th percentiles (lower and upper hinge respectively) are represented as boxes. Outside values are not displayed in the graphs. Differences between timepoints were assessed by Friedman’s test, followed by two-by-two comparisons corrected with Bonferroni’s test for multiple comparisons

In all the cohorts, CHMI was associated with an expansion of rcMBCs, which occurred sooner (D11) in the vaccinated and semi-immune groups (Fig. 4A–C). This was accompanied by an expansion of PCGCs and a decrease of naïve B cells in the semi-immune cohort (Fig. 4C). With regards to the expression of markers, an expansion of raMBCs-PD1^+^ and active naive-PD1^+^ B cells was observed in the naïve and vaccinated groups, respectively (Fig. 4A andB). Finally, we observed an increase in the percentage of several MBCs producing IgG^+^ from day 0 to day 11 in the semi-immune group (Fig. 4C). Non-significant results are not shown.

**Fig. 5:**
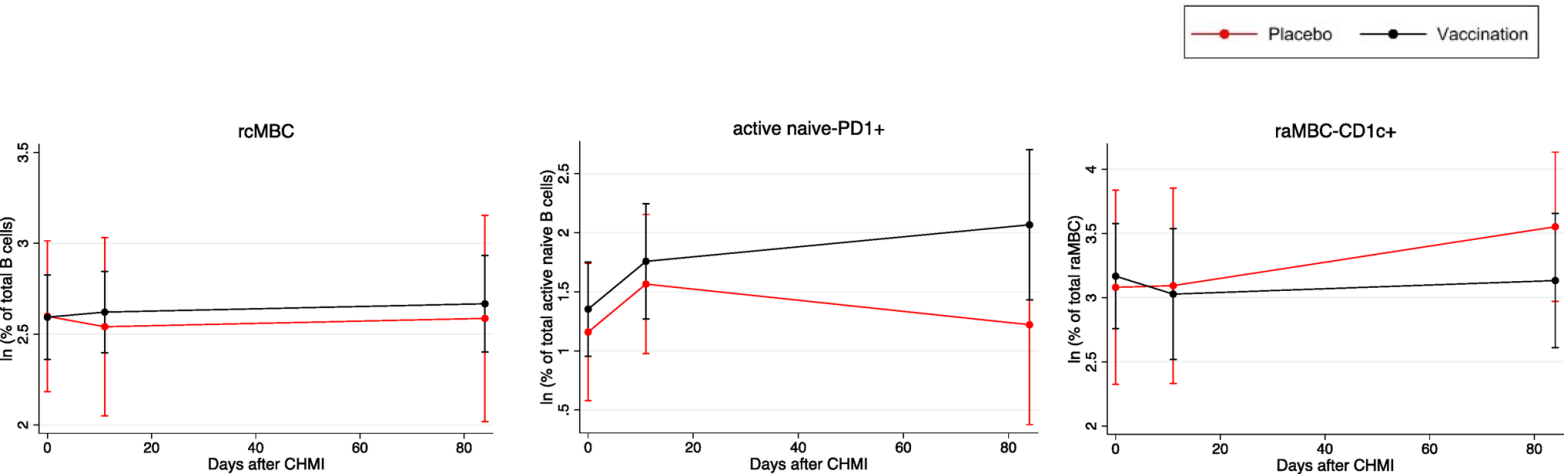
Effect of vaccination status in the B cell distribution at different follow-up periods after CHMI. Mean cell subset frequencies plus standard error of the mean are represented for the different arms of the TÜCHMI-2 cohort over time. Only B cell populations with statistically significant interactions with vaccination are shown

Next, the effect of the interaction of previous vaccination with the average B cell changes during follow-up was assessed in the TÜCHMI-002 cohort. There was a significant (or borderline significant) and positive effect of vaccination for rcMBCs (β = 0.190, 95% CI 0.011–0.368, *p* = 0.037) (Fig. 5) at D11, and for active naïve-PD1^+^ (β = 0.637, 95% CI 0.058 to 1.217, p = 0.031) (Fig. 5), acMBC-IgG^+^ (β = 0.166, 95%CI − 0.025 to 0.358, *p* = 0.088) and PCGCs (β = 0.874, 95%CI − 0.137 to 1.884, *p* = 0.090) at D84. In addition, a negative effect was observed for CD1c^+^raMBC (β = − 0.328, 95%CI − 0.621 to − 0.032, *p* = 0.030) (Fig. 5) at D84 and CD1c^+^ naive B cells (β = − 0.201, 95% CI − 0.408 to 0.006, *p* = 0.058). These results did not change when we adjusted this analysis for malaria infection occurring after the CHMI: rcMBCs, β = 0.188, 95% CI 0.010 to 0.367, p = 0.038 at D11; active naïve-PD1^+^, β = 0.640, 95%CI 0.062 to 1.219, *p* = 0.030 at D84; acMBC-IgG^+^, β = 0.166, 95% CI − 0.026 to 0.359, *p* = 0.090 at D84; PCGCs, β = 0.890, 95% CI − 0.127 to 1.908, *p* = 0.087 at D84; CD1c^+^raMBC, β = − 0.328, 95% CI − 0.623 to − 0.034, *p* = 0.029 at D84; CD1c^+^ naive B cells, β = − 0.201, 95% CI − 0.409 to 0.006, *p* = 0.057 at D84.

Furthermore, the interaction of the sickle cell trait (HbAS) with the average B cell changes after the CHMI was assessed in the LACHMI-001 cohort. During the follow-up, semi-immune individuals with sickle cell trait had more aaMBCs (β = 1.244, 95% CI 0.041 to 2.446, *p* = 0.043) and raMBCs (β = 0.752, 95% CI 0.036 to 1.467, *p* = 0.039) at D5 compared to participants with normal hemoglobin (HbAA). On the contrary, sickle cell trait had a negative effect on acMBC-PD1^+^ (β = − 0.853, 95% CI − 1.395 to − 0.310, *p* = 0.002), raMBC-PD1^+^ (β = − 0.926, 95% CI − 1.445 to − 0.406, *p* < 0.001) and rcMBC-PD1^+^ (β = − 0.962, 95% CI − 1.679 to − 0.245, *p* = 0.009) frequencies. These results were maintained after adjusting for malaria infection post-CHMI (all results refer to day 5): aaMBCs, β = 1.233, 95% CI 0.045 to 2.442, *p* = 0.042; raMBCs, β = 0.731, 95% CI 0.024 to 1.436, *p* = 0.043; acMBC-PD1^+^, β = − 0.846, 95% CI − 1.386 to − 0.305, *p* = 0.002; raMBC-PD1^+^, β = − 0.919, 95% CI − 1.442 to − 0.394, *p* = 0.001; and rcMBC-PD1^+^, β = − 0.955, 95% CI − 1.669 to − 0.242, *p* = 0.009.

### Predictors of infection after CHMI

**Fig. 6:**
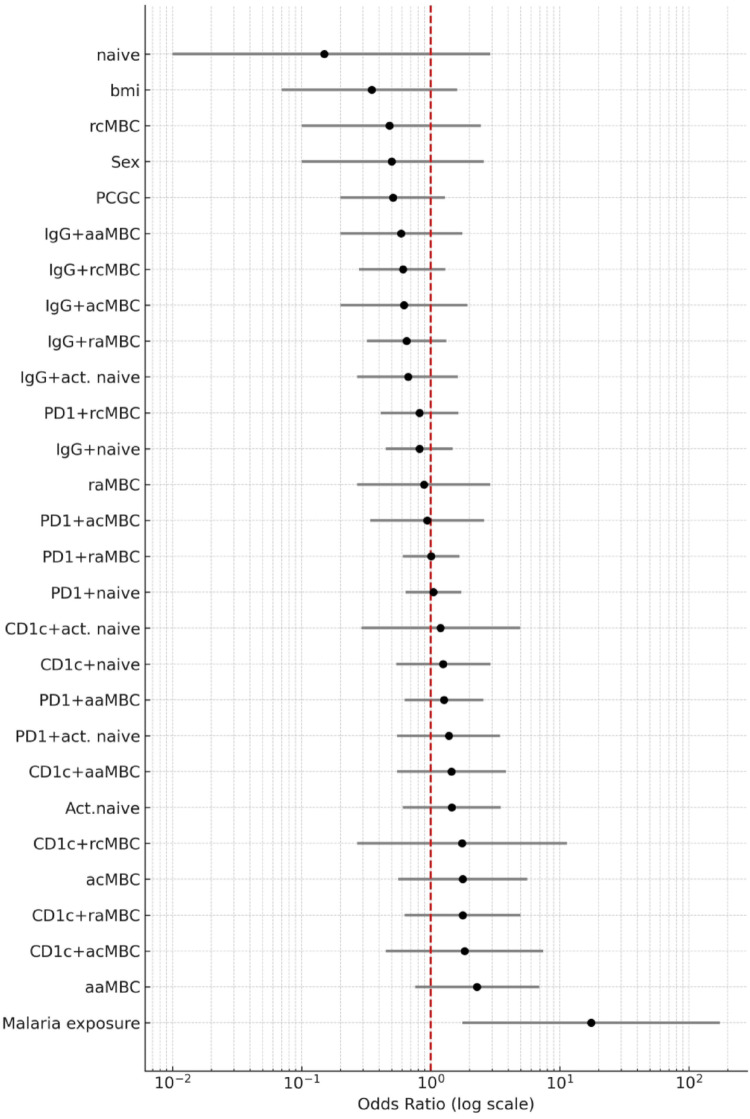
Forest plot showing odds ratios (ORs) and corresponding 95% confidence intervals for the associations of the B cell frequencies and other variables measured baseline with malaria infection after CHMI. The ORs were estimated by means of individual logistic regressions with the samples from LACHMI-001 and the PfSPZ-CVac-vaccinated group (n = 29). The vertical red dashed line indicates the null value (OR = 1.0), representing no association with the outcome

**Fig. 7:**
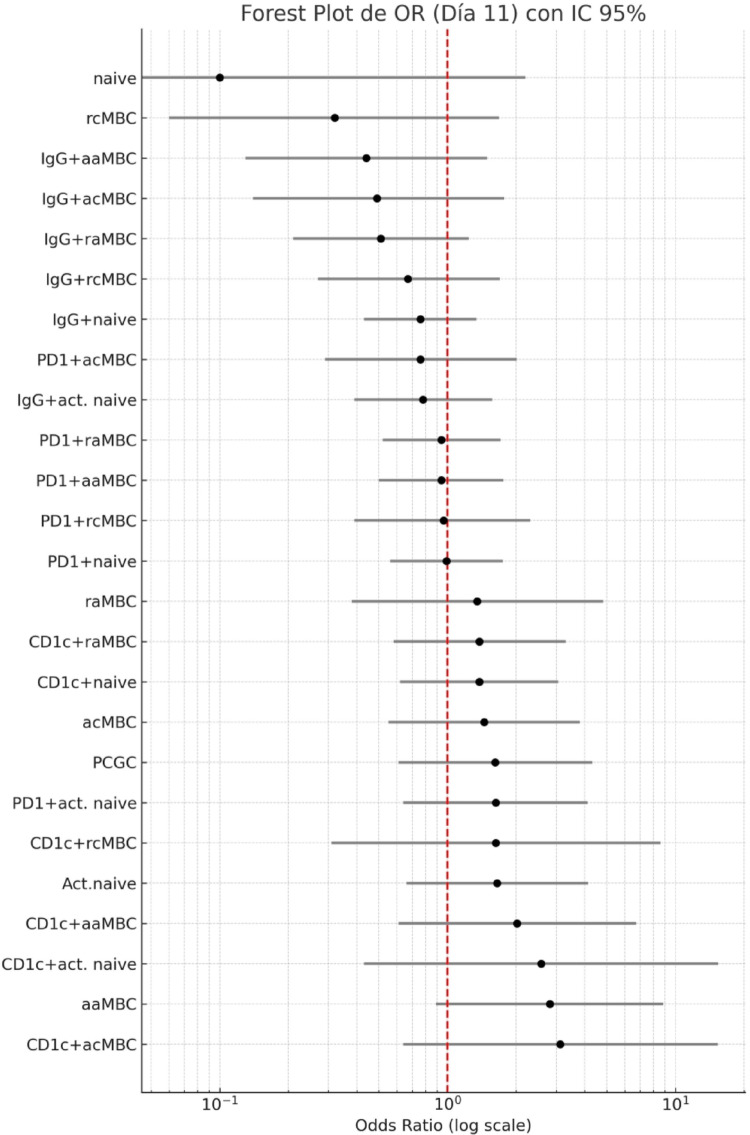
Forest plot showing odds ratios (ORs) and corresponding 95% confidence intervals for the associations of the B cell frequencies at day 11 with malaria infection after CHMI. The ORs were estimated by means of individual logistic regressions with the samples from LACHMI and the PfSPZ-CVac-vaccinated group (n = 29). The vertical red dashed line indicates the null value (OR = 1.0), representing no association with the outcome

This was evaluated in the vaccinated and semi-immune groups together. Semi-immune donors had 17 × higher risk of having an infection after the CHMI than vaccinated donors, but none of the B cell subsets in their baseline levels were significantly associated with infection after CHMI (Supplementary Table 3 and Fig. 6). At D11, aaMBC frequency had a borderline non-significant positive association with infection (Supplementary Table 3 and Fig. 7). However, after adjusting for previous malaria exposure, the association was lost (OR = 2.45, 95% CI 0.67 to 8.94, *p* = 0.172). When we performed this analysis separately in the vaccinated and semi-immune groups, no associations of baseline or D11 B cells levels and infection were found (data not shown).

### Relationship between B cells, Pf antibody levels, and cytokine concentrations

**Fig. 8:**
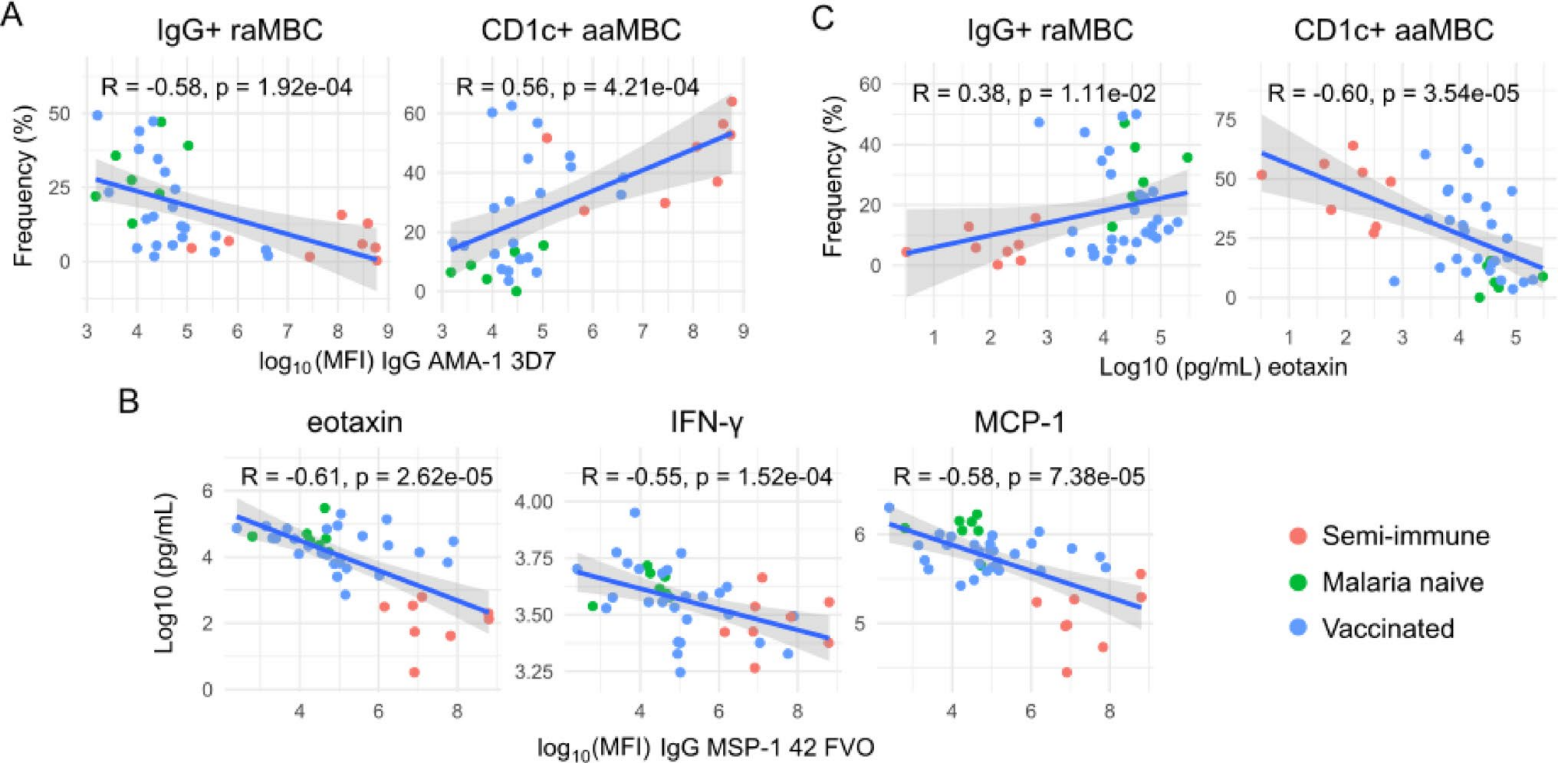
Significant correlations of B cell frequencies, *P. falciparum* IgG levels (surrogates of Pf exposure) and cytokine concentrations at baseline. Scatter plots of immune responses measured in semi-immune (LACHMI-001, red), vaccinated (TÜCHMI-002, blue dots) and naïve (TÜHMI-001, green) individuals together, with Spearman’s coefficients and raw p-values. **A** IgG to AMA-1 correlated negatively with IgG^+^ resting atypical memory B cells (raMBC) and positively with CD1c^+^ active atypical memory B cells (aaMBC). **B** Negative correlation of IgG to MSP1_42_ vs. eotaxin, IFN-γ or MCP-1. **C** Eotaxin correlated negatively with CD1c^+^ aaMBC and positively with IgG^+^ raMBC

A negative correlation was found between anti-IgG to Pf antigens that are well known markers of exposure (particularly PfMSP-1_19_, PfMSP-1_42_, PfAMA-1) and raMBC or rcMBC frequencies, in all individuals together, at baseline (rho range − 0.61 to − 0.25, raw *p* < 0.05, Figure S1A) and all time points together (rho range − 0.44 to − 0.1, raw *p* < 0.05, Figure S1B). In contrast, a positive association was observed with active naïve B cell proportions (baseline rho range 0.4–0.24, all timepoints together rho range 0.41–0.19, raw *p* < 0.05). In addition, a moderate negative correlation was found between Pf antibody levels (mostly PfMSP-1s and PfAMA-1s) and IgG^+^ B cell frequencies, while a positive moderate correlation occurred between such antibodies and CD1c^+^ B cell frequencies (Fig. 8A, Figure S1). Similar results were observed at D11 after the CHMI but not at D84 (data not shown). However, from these cell subsets only rcMBC at baseline correlated with increased fold-changes in IgG levels against PfPTRAMP, PfAARP, PfRh5, PfCelTOS, and LSA-1 from baseline to D84 (Fig S2B). Similarly, acMBC at baseline positiverly correlated with IgG fold-changes against 11 Pf antigens to D11 (Fig S2A). In contrast, naïve B cells correlated negatively with IgG fold-changes up to D11 (mainly for markers of Pf exposure) and D84 (for non-exposure markers).

The antibody markers of Pf exposure PfMSP-1_19_, PfMSP-1_42_, PfAMA-1, PfEXP-1, PfEBA-175 also correlated negatively with the concentrations of eotaxin, MCP-1, IP-10, IFN-γ and other cytokines when considering all groups and time points together, but mostly at baseline (Fig. 8B, Figure S3). In turn, these cytokines/chemokines (mostly eotaxin) correlated moderately and negatively with the frequencies of CD1c^+^ B cells, and positively with IgG^+^ B cells (Fig. 8C, Figure S4). Cytokine levels at baseline reflect the immunological status of the participants, that is the baseline level of immune activity, inflammation and preparedness against stimuli.

**Fig. 9:**
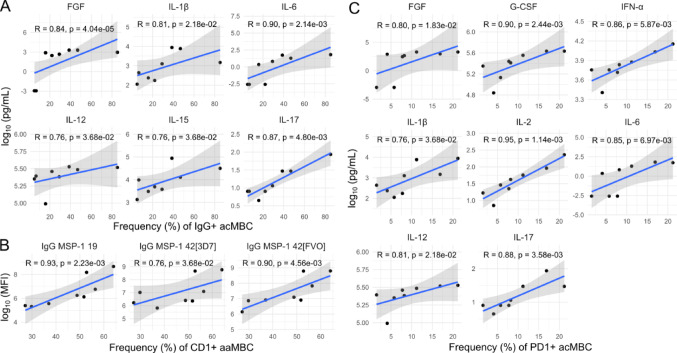
Significant correlations of B cell frequencies, *P. falciparum* IgG levels (surrogates of Pf exposure) and cytokine concentrations at baseline in semi-immune individuals. **A** Positive correlations of IgG^+^ MBC) vs. pro-inflammatory cytokines in semi-immune volunteers. **B** CD1c^+^ active atypical MBC (aaMBC) correlated positively with anti-PfMSP1_19_ and anti-PfMSP1_42_ IgG levels. **C** PD1^+^ active classical MBC (acMBC) correlated positively with pro-inflammatory cytokines. Full correlograms of all immune markers are shown in supplementary materials

Collectively, data show that higher Pf exposure, manifested by higher anti-Pf IgG levels (most prominent in the semi-immune), correlated with lower eotaxin and IFN related cytokines, as well as higher frequencies of CD1c^+^ B cells and lower frequencies IgG^+^ B cells. These correlations, however, were driven mostly by the semi-immune group. In LACHMI-001 semi-immune individuals alone, IgG^+^ acMBC correlated positively with pro-inflammatory cytokines (IL-1, IL-6, IL-12, IL-15, IL-17, FGF; Fig. 9A) and negatively with regulatory cytokines (IL-10, IL-13) (Figure S5), while CD1c^+^ aaMBC correlated strongly and positively with anti-PfMSP1_19_ and anti-PfMSP1_42_ IgG levels (Fig. 9B). PD1^+^ acMBC also correlated positively with several pro-inflammatory cytokines including IFN-α, IL-1β, IL-6, IL-12, IL-17 and IL-2 in semi-immune participants (Fig. 9C). In those individuals, the frequency of aaMBCs correlated negatively with eotaxin levels, but this result did not reach statistical significance (Figure S5).

## Discussion

Before the CHMI and compared to naïve individuals, semi-immune but not vaccinated individuals had increased frequencies of immature and active naïve B cells, as well as active classical and atypical MBCs. First acute malaria episodes have been associated with an expansion of plasmablast cells, atypical MBCs and/or naïve B cells [29, 44], however the frequencies may decline to baseline levels 35 days after a single malaria infection. This suggests that multiple rather than single malaria exposures are necessary for durable changes in B cell profiles, consistent with our findings. Interestingly, baseline frequencies of CD1c^+^ cells within each B cell subset, were higher in the semi-immune compared to naïve individuals, correlated negatively with eotaxin and positively with anti-Pf antibodies, the latter well-known markers of malaria exposure [45]. CD1c expression in dendritic cells has been positively associated to Pf-malaria exposure and protection [46, 47]. However, to the best of our knowledge, the expression of this marker on B cells in relation to malaria has not been previously explored and more studies are necesary to confirm whether it is a marker of exposure. On the other hand, the percentages of IgG^+^ cells were lower in semi-immune than naive donors, for most B cell subsets and, accordingly, a negative correlation of IgG^+^ cells with anti-Pf antibody levels was found. Of note, the decreased frequency of IgG^+^ was not detected for the expanded aaMBC population. This negative association between IgG^+^ cell frequencies and malaria exposure is in contrast with previous results observed in a cohort of pregnant and non-pregnant individuals from Papua New Guinea [24, 48]. But pregnancy itself, exposure to both Pf and *P. vivax* malaria or other population factors rather than malaria exposure may be responsible for that finding.

CHMI resulted in an expansion of rcMBCs in all groups, and of IgG^+^ MBCs at D11 in the semi-immune group, as expected after an infection (reviewed in [49]). Besides, an expansion of certain PD1^+^ B cell populations occurred after the CHMI. We and others have previously demonstrated that malaria exposure/infection is associated with elevated frequencies of PD1^+^ B cells compared to healthy individuals [37, 48]. However, in this study no correlation was found between PD1^+^ MBCs and anti-Pf antibody levels. The function of PD1 is not yet much recognized on B cells, whilst it is a well-known inhibitory marker in T cells, associated largely to malaria infection/exposure [50–52], also in NK cells [53]. Although it is classically considered that PD1^+^ T cells are exhausted and associated with impaired parasite control and infection chronicity [54], some studies have reported an active role for leukocytes expressing this marker [53, 55]. Indeed, PD1 is expressed in activated cells and is involved in immune-homeostatic mechanisms. Furthermore, PD1^+^ acMBC were positively correlated with inflammatory cytokines in semi-immune individuals suggesting PD1 as a marker of immune activation. In consonance with all these results, previous vaccination with PfSPZ-CVac was associated with increased percentages of rcMBCs and PD1^+^ active naïve B cells over post-CHMI follow-up, compared to placebo treatment. This is of relevance because it suggests that B-cell responses are boosted in vaccine recipients during infection without affecting malaria-related alterations in B-cell subsets, such as atypical MBCs, which may be associated with tolerogenic responses. Unfortunately, limited sample size when stratifying by vaccine dose did not allow us to assess dose effect modifications.

One-third of the population estimated to have the sickle cell trait lives in sub-Saharan Africa, and children with HbAS have decreased susceptibility to clinical malaria [56]. Among the potential explanations for this protection are: (i) enhanced naturally-acquired immunity to malaria, (ii) impaired growth of Pf on HbAS-erythrocytes or increased splenic removal, and (iii) altered surface expression of cytoadherence proteins on infected HbAS-erythrocytes [57]. In our CHMI study, an increase of atypical MBC frequencies was observed at D5 on individuals with the HbAS, and a decrease of PD1^+^ MBCs, even after adjusting for current infection status. Antibody analysis including all semi-immune participants (N = 20) from the same study (LACHMI-001) showed that sickle cell trait individuals compared with the semi-immune with normal hb had significantly lower IgM and IgG4 levels, and a trend of higher IgG1 and IgG3 response against certain pre-erythrocytic and blood stage antigens [Gómez-Pérez et al. [41]]. To the best of our knowledge, the B cell phenotype change after malaria (or any other) infection has not been described in carriers of the sickle cell trait, and more studies are necessary to confirm these findings and the associated antibody response, especially considering our restricted sample size.

The negative correlation of Pf antibodies with eotaxin is in line with the significantly lower eotaxin levels found in lifelong malaria exposed semi-immune individuals compared to the vaccinated or naïve groups (Moncunill et al., in preparation). Other cytokines related to IFN responses were also negatively correlated with exposure, suggesting a broad impact of cumulative malaria episodes on the immune profile of individuals towards a tolerogenic profile. Consistently these cytokines were negatively associated with B cell subsets expressing CD1c, which increased frequencies were linked to malaria exposure. Ex vivo studies show that human B cells-activation via BCR crosslinking, but not by CD40L alone (T-cell help), upregulate CD1c in naïve, memory and marginal zone-like B cells, and that elevated CD1c enhanced their function as antigen presenting cells [38]. Hence, CD1c expression might be a marker of B cell activation via BCR crosslinking by Pf antigens, and could be related to a regulatory and tolerogenic role given the observed negative correlation with IFN related cytokines. Accordingly, the semi-immune, especially individuals with sickle cell trait, were asymptomatic or had less malaria symptoms than the malaria-naïve [32]. However, we did not find the previously observed negative correlation between eotaxin and atypical MBCs in endemic populations [24]. This may be explained because in this study individuals with long and short exposure to malaria were included, and the semi-immune cohort was actually too small (n = 8) to observe significant differences. Nevertheless, in those individuals, frequencies of MBC expressing IgG, diminished by exposure with the exception of aaMBC, were positively correlated with cytokines related to inflammation, Th1 and Th17 responses, suggesting a more tolerogenic and blunt immune status at baseline related with the decreased frequencies of these B cell subsets.

This study is limited by the small sample size of the cohorts and the need to treat individuals at first detection of parasites, which might have influenced the natural immune response. Another limitation is the lack of antigen-specific B cell data, although selection of Pf antigens for that purpose would be challenging. In addition, cellular samples were analyzed in three different periods of time, which may have hampered comparability. Nevertheless, important strengths of our study include its novelty, being the first to analyze the B cell phenotype dynamics as a response to CHMI in individuals with different immunity and hb status, as well as the analysis of B cell surface markers not previously studied in the context of malaria such as CD1c. Still, we cannot discard that some of the differences observed between African and European volunteers are due to different genetic background.

In summary, cumulative but not a single (vaccine) malaria exposure was associated with increased frequencies of many B lymphocyte subsets, with higher and lower percentages of CD1c and IgG expressing cells, respectively, and a decrease of circulating cytokines previously linked to altered MBC phenotypes, mostly eotaxin, overall suggesting a tolerogenic profile. The CHMI was associated with an early expansion of rcMBC in all three cohorts, and this was increased in the vaccine- compared to the placebo-recipients, suggesting boosting of B cell memory in the vaccinated. The effect of CHMI on B cell phenotypes in semi-immune individuals was also modified by the sickle cell trait. The correlation of these B cell subsets with anti-Pf antibodies suggests that this effect is truly driven by the intensity of exposure to Pf parasites and supports further studies to assess the impact of those changes on the humoral response and naturally acquired immunity against malaria.

## Supplementary Information

Below is the link to the electronic supplementary material.

Supplementary Material 1

## Data Availability

The data that support the findings of this study are available from the corresponding author upon reasonable request. The data will be shared after a consideration of the request, ensuring that the purpose aligns with the ethical guidelines and the informed consent obtained from study participants. Data will be provided in a de-identified format to ensure participant confidentiality.
