# Supplementary material for: Effect of controlled human Plasmodium falciparum infection on B cell subsets in individuals with different levels of malaria immunity
Source: Med Microbiol Immunol. 2025 Sep 27;214(1):47. doi: 10.1007/s00430-025-00847-x (PMC12476306; doi:10.1007/s00430-025-00847-x)
Supplement: Supplementary file 1 — Supplementary Material 1 [file 430_2025_847_MOESM1_ESM.docx]

### SUPPLEMENTARY INFORMATION

**Effect of controlled human *Plasmodium falciparum* infection on B cell subsets in individuals with different levels of malaria immunity**

Pilar Requena^1,2,3,#^, Gloria Patricia Gómez-Pérez^4,5^, Matthew B. B. McCall^4,6,7^, Diana Barrios^4^, Ruth Aguilar^4^, Julia Fernández-Morata^4^, Marta Vidal^4^, Joseph J. Campo^8^, Carla Sanchez^4^, Maria Yazdabankhsh^9^, B. Kim Lee Sim^1^, Stephen L. Hoffman^10^, Peter Kremsner^11,12^, Bertrand Lell^11,13^, Benjamin Mordmüller^7,12,14^, Carlota Dobaño^4,5,14*^, Gemma Moncunill^4,5,14,#*^

1. Universidad de Granada, Departamento de Medicina Preventiva y Salud Pública, Granada, Spain.

2. Instituto de Investigación Biosanitaria de Granada (ibs.GRANADA), Granada, Spain.

3. Centro de Investigación Biomédica en Red de Epidemiología y Salud Pública (CIBERESP), Madrid, Spain.

4. ISGlobal, Barcelona, Catalonia, Spain.

5. Facultat de Medicina i Ciències de la Salut, Universitat de Barcelona, Barcelona, Spain.

6. Department of Medical Microbiology and Infectious Diseases, Erasmus MC, Rotterdam, The Netherlands.

7. Department of Medical Microbiology, Radboud Center for Infectious Diseases, Radboud University Medical Center, Geert Grooteplein Zuid 28, 6525 GA, Nijmegen, The Netherlands.

8. Antigen Discovery, Irvine, CA, USA

9. Leiden University Center for Infectious Diseases (LUCID), Leiden University Medical Center, ZA, Leiden, Netherlands.

10. Sanaria Inc., Rockville, MD, USA.

11. Centre de Recherches Médicales de Lambaréné (CERMEL), Gabon.

12. Institute of Tropical Medicine and German Center for Infection Research, University of Tübingen, Tübingen, Germany.

13. Department of Medicine I, Division of Infectious Diseases and Tropical Medicine, Medical University of Vienna, Vienna, Austria.

14. CIBER de Enfermedades Infecciosas (CIBERINFEC), Barcelona, Spain.

*shared

# Correspondence:

Pilar Requena, Facultad de Farmacia, Departamento de Medicina Preventiva y Salud Pública. Campus de Cartuja s/n 18170, Granada (Spain). ORCID: 0000-0002-2298-4085

Email: prequena@ugr.es

Gemma Moncunill, ISGlobal, C/Rosselló, 132, 08036 Barcelona (Spain). ORCID: 0000-0001-5105-9836

Email: gemma.moncunill@isglobal.org

### Supplementary Table 1. Antibodies used in the multiparameter flow cytometry panel

| **Antibody** | **Fluorocrome** | **Company** | **Cat. No.** | **Clone** |
| --- | --- | --- | --- | --- |
| anti-CD3 | Horizon v500 | BD | 561416 | UCHT1 |
| anti-CD16 | Horizon v500 | BD | 561394 | 3G8 |
| anti-CD14 | Horizon v500 | BD | 561391 | M5e2 |
| anti-CD19 | PE/CF594 | BD | 562321 | HIB19 |
| anti-IgD | APC/H7 | BD | 561305 | IA6-2 |
| anti-CD27 | APC | BD | 558664 | M-T271 |
| anti-CD21 | FITC | Beckman Coulter | PN IM0473U | BL13 |
| anti-CD38 | PerCP | BioLegend | 303520 | HIT2 |
| anti-CD10 | BV421 | BioLegend | 312217 | HI10a |
| anti-surface IgM | PE | BD | 555783 | G20-127 |
| anti-surface IgG | PE/Cy7 | BD | 561298 | G18-145 |
| anti-PD1 | BV605 | BioLegend | 329923 | EH12.2H7 |
| anti-CD1c | AF700 | BioLegend | 331530 | L161 |

**Supplementary table 2.** Differences in frequencies of B cells between groups at baseline.

| Cell subset | Parental cell population | Naïve (n=19) | Vaccinated (n=21) | Semi-immune (n=8) | p-value  naïve vs vacc | p-value  naïve vs semi | p-value  vacc vs semi |
| --- | --- | --- | --- | --- | --- | --- | --- |
| Immature | VBC | 5.5 (3.3-9.6) | 7.1 (3.6-11.8) | 12.1 (7.3-16.1) | 0.589 | **0.049** | 0.199 |
| PCGC | Non-immature VBC | 0.9 (0.4-1.6) | 0.7 (0.4-1.3) | 0.4 (0.3-0.6) | 0.202 | **0.037** | 0.384 |
| aaMBC | Switched | 2.1 (0.7-3.7) | 2.4 (1.3-3.6) | 3.9 (2.9-4.4) | 0.911 | **0.075** | 0.167 |
| acMBC | Switched | 2.1 (0.9-3.5) | 1.8 (1.2-3.2) | 3.8 (2.6-5.2) | 1.000 | **0.079** | **0.085** |
| raMBC | Switched | 10.1 (7.5-16.8) | 12.7 (7.1-14.4) | 4.7 (5.6-9.2) | 1.000 | 0.100 | 0.187 |
| rcMBC | Switched | 21.1 (11.0-28.5) | 14.5 (11.7-16.4) | 10.4 (7.7-16.2) | 0.311 | 0.109 | 0.586 |
| act. naive | Unswitched | 1.48 (0.8-3.0) | 2.0 (1.4-3.3) | 3.4 (2.6-8.4) | 0.626 | **0.009** | **0.047** |
| naive | Unswitched | 41.4 (35.7-51.5) | 49.4 (36.7-53.2) | 43.4 (37.9-50.4) | 0.502 | 1.000 | 0.716 |
| CD1c^+^aaMBC | aaMBC | 13.9 (8.1-24.9) | 25.0 (10.8-42.0) | 50.25 (33.4-54.6) | 0.251 | **0.002** | **0.037** |
| CD1c^+^acMBC | acMBC | 33.5 (21.1-57.3) | 43.0 (23.3-67.1) | 70.9 (50.5-74.9) | 0.629 | **0.038** | 0.149 |
| CD1c^+^raMBC | raMBC | 22.4 (6.2-43.8) | 23.2 (14.7-44.3) | 60.4 (45.5-69.7) | 0.724 | **0.002** | **0.009** |
| CD1c^+^rcMBC | rcMBC | 51.0 (29.1-69.9) | 63.6 (36.8-73.5) | 74.5 (60-78.5) | 0.555 | **0.072** | 0.285 |
| CD1c^+^act. naive | Act. naive | 26.3 (16.4-42.0) | 38.6 (23.6-59.3) | 61.95 (45.0-71.7) | 0.282 | **0.004** | **0.066** |
| CD1c^+^naive | Naive | 16.4 (4.6-36.1) | 17.8 (9.8-50.1) | 58.3 (42.5-67.5) | 0.517 | **0.002** | **0.018** |
| PD1^+^aaMBC | aaMBC | 4.49 (3.8-9.7) | 5.4 (3.9-12.7) | 14.7 (5.7-21.6) | 0.540 | **0.050** | 0.216 |
| PD1^+^acMBC | acMBC | 4.4 (3.6-6.8) | 5.5 (3.1-9.0) | 8.0 (4.9-14.0) | 0.637 | 0.151 | 0.446 |
| PD1^+^raMBC | raMBC | 0.3 (0.2-1.2) | 0.3 (0.2-1.5) | 0.9 (0.4-1.4) | 0.342 | 0.296 | 1.000 |
| PD1^+^rcMBC | rcMBC | 0.9 (0.6-1.7) | 1.5 (0.9-4.0) | 2.4 (1.4-3.8) | 0.129 | 0.143 | 1.000 |
| PD1^+^act. naive | Act. naive | 3.0 (1.3-6.3) | 4.6 (1.8-6.5) | 6.3 (3.4-10.1) | 0.892 | 0.108 | 0.241 |
| PD1^+^naive | Naive | 0.3 (0.2-.0.7) | 0.4 (0.3-1.8) | 0.9 (0.6-2.1) | 0.741 | 0.100 | 0.260 |
| IgG^+^aaMBC | aaMBC | 36.3 (25.9-43.4) | 26.3 (20.1-37.9) | 45.35 (21.1-55.7) | **0.085** | 0.905 | **0.072** |
| IgG^+^acMBC | acMBC | 48.2 (37.7-5.9) | 43.7(29.2-55.0) | 25.5 (11.2-42.7) | 0.426 | **0.030** | 0.183 |
| IgG^+^raMBC | raMBC | 22.9 (12.8-39.1) | 11.9 (5.3-23.4) | 5.2 (3.0-9.8) | **0.045** | **0.001** | 0.100 |
| IgG^+^rcMBC | rcMBC | 39.5 (23.2-54.5) | 36.4 (11.6-43.4) | 12.2 (5.0-15.25) | 0.299 | **0.006** | **0.075** |
| IgG^+^act. naive | Act. naive | 17.5 (8.8-33.3) | 10.8 (7.7-14.8) | 11.7 (4.7-16.8) | 0.110 | 0.170 | 1.000 |
| IgG^+^naive | Naive | 10.4 (6.9-43.2) | 9.0 (4.5-13.6) | 3.4 (1.9-5.6) | 0.370 | **0.002** | **0.027** |

Median plus interquartile range of cellular percentages are shown in the cells. P-value corresponds to Dunn’s test corrected for multiple comparisons with the Bonferroni method. Highlighted in bold if p-value <0.1.

**Supplementary Table 3. Predictors of malaria infection**

| Variables | Baseline | | | | | Day 11 | | | | |
| --- | --- | --- | --- | --- | --- | --- | --- | --- | --- | --- |
|  | OR | 95% CI | | | p-value | OR | 95% CI | | | p-value |
| Malaria exposure  (semi- vs vacc(ref)) | **17.50** | **1.76** | **–** | **174.42** | **0.015** | - | - | | | - |
| Sex (wom vs men (ref)) | 0.50 | 0.10 | – | 2.58 | 0.407 | - | - | | | - |
| bmi (≥25 vs <25(ref)) | 0.35 | 0.07 | – | 1.61 | 0.176 | - | - | | | - |
| aaMBC | 2.29 | 0.76 | – | 6.92 | 0.142 | **2.81** | **0.89** | **–** | **8.84** | **0.077** |
| acMBC | 1.77 | 0.56 | – | 5.59 | 0.330 | 1.45 | 0.55 | – | 3.81 | 0.447 |
| rcMBC | 0.48 | 0.10 | – | 2.44 | 0.380 | 0.32 | 0.06 | – | 1.68 | 0.178 |
| raMBC | 0.89 | 0.27 | – | 2.89 | 0.848 | 1.35 | 0.38 | – | 4.83 | 0.647 |
| PCGC | 0.51 | 0.20 | – | 1.29 | 0.156 | 1.62 | 0.61 | – | 4.32 | 0.335 |
| Act.naive | 1.46 | 0.61 | – | 3.50 | 0.395 | 1.65 | 0.66 | – | 4.15 | 0.287 |
| naive | 0.15 | 0.01 | – | 2.89 | 0.211 | 0.10 | 0.00 | – | 2.20 | 0.143 |
| IgG^+^aaMBC | 0.59 | 0.20 | – | 1.76 | 0.342 | 0.44 | 0.13 | – | 1.49 | 0.189 |
| IgG^+^acMBC | 0.62 | 0.20 | – | 1.93 | 0.406 | 0.49 | 0.14 | – | 1.77 | 0.276 |
| IgG^+^raMBC | 0.65 | 0.32 | – | 1.32 | 0.234 | 0.51 | 0.21 | – | 1.24 | 0.137 |
| IgG^+^rcMBC | 0.61 | 0.28 | – | 1.30 | 0.199 | 0.67 | 0.27 | – | 1.70 | 0.403 |
| IgG^+^act. naive | 0.67 | 0.27 | – | 1.63 | 0.376 | 0.78 | 0.39 | – | 1.57 | 0.486 |
| IgG^+^naive | 0.82 | 0.45 | – | 1.48 | 0.505 | 0.76 | 0.43 | – | 1.34 | 0.337 |
| PD1^+^aaMBC | 1.27 | 0.63 | – | 2.56 | 0.500 | 0.94 | 0.50 | – | 1.76 | 0.843 |
| PD1^+^acMBC | 0.94 | 0.34 | – | 2.59 | 0.902 | 0.76 | 0.29 | – | 2.01 | 0.580 |
| PD1^+^raMBC | 1.01 | 0.61 | – | 1.67 | 0.979 | 0.94 | 0.52 | – | 1.71 | 0.850 |
| PD1^+^rcMBC | 0.82 | 0.41 | – | 1.64 | 0.566 | 0.96 | 0.39 | – | 2.31 | 0.920 |
| PD1^+^act. naive | 1.38 | 0.55 | – | 3.45 | 0.491 | 1.63 | 0.64 | – | 4.12 | 0.304 |
| PD1^+^naive | 1.05 | 0.64 | – | 1.73 | 0.846 | 0.99 | 0.56 | – | 1.75 | 0.977 |
| CD1c^+^aaMBC | 1.45 | 0.55 | – | 3.84 | 0.458 | 2.02 | 0.61 | – | 6.72 | 0.252 |
| CD1c^+^acMBC | 1.84 | 0.45 | – | 7.48 | 0.397 | 3.12 | 0.64 | – | 15.32 | 0.160 |
| CD1c^+^raMBC | 1.77 | 0.63 | – | 4.96 | 0.276 | 1.38 | 0.58 | – | 3.30 | 0.471 |
| CD1c^+^rcMBC | 1.75 | 0.27 | – | 11.40 | 0.560 | 1.63 | 0.31 | – | 8.60 | 0.567 |
| CD1c^+^act. naive | 1.19 | 0.29 | – | 4.93 | 0.810 | 2.58 | 0.43 | – | 15.37 | 0.299 |
| CD1c^+^naive | 1.25 | 0.54 | – | 2.91 | 0.599 | 1.38 | 0.62 | – | 3.06 | 0.433 |

The association of the B cell frequencies and other variables with malaria infection after CHMI was assessed by means of individual logistic regressions with the samples from LACHMI and the PfSPZ-CVac-vaccinated group (n=29), estimating the odds ratios (OR) and 95% confidence intervals (CI).

**Supplementary Figures**

**A**

**B**

**Figure S1. Spearman’s correlation matrices of frequencies of B cell subpopulations and B cells expressing IgG, PD1 and CD1c markers and anti-Pf antibody levels in all individuals**. The matrices represent the correlations of these immune variables measured in blood of semi-immune (LACHMI-001), vaccinated (TÜCHMI-002), and naïve (TÜCHMI-001) individuals, collected before the CHMI (A) or at all timepoints together (B). Colour of the cells represent the Spearman’s rho value, ranging from -1 to +1. * p<0.05, ** p<0.01, *** p<0.001.

**A**

**B**

**Figure S2. Spearman’s correlation matrices of frequencies of B cell subpopulations and B cells expressing IgG, PD1 and CD1c markers and fold-change increases of anti-Pf antibody levels in all individuals**. The matrices represent the correlations of these immune variables measured in blood of semi-immune (LACHMI-001), vaccinated (TÜCHMI-002), and naïve (TÜCHMI-001) individuals with IgG fold-change increases from baseline to D11 (A) or to D84 (B). Colour of the cells represent the Spearman’s rho value, ranging from -1 to +1. * p<0.05, ** p<0.01, *** p<0.001.

**A**

**B**

**Figure S3. Spearman’s correlation matrices of anti-Pf antibody and cytokine levels in all individuals**. The matrices represent the correlations of these immune variables measured in blood of semi-immune (LACHMI-001), vaccinated (TÜCHMI-002), and naïve (TÜCHMI-001) individuals, collected before the CHMI (A) or at all timepoints together (B). Colour of the cells represent the Spearman’s rho value, ranging from -1 to +1. * p<0.05, ** p<0.01, *** p<0.001.

**A**

**B**

**Figure S4. Spearman’s correlation matrices of frequencies of B cell subsets expressing IgG, PD1 and CD1c markers and cytokine concentrations in all individuals**. The matrices represent the correlations of these immune variables measured in blood of semi-immune (LACHMI-001), vaccinated (TÜCHMI-002), and naïve (TÜCHMI-001) individuals, collected before the CHMI (A) or at all timepoints together (B). Colour of the cells represent the Spearman’s rho value, ranging from -1 to +1. * p<0.05, ** p<0.01, *** p<0.001.

**A**

**B**

**Figure S5. Spearman’s correlation matrices of frequencies of B cell subsets expressing IgG, PD1 and CD1c markers and cytokine concentrations in semi-immune individuals**. The matrices represent the correlations of these immune variables measured in blood of semi-immune (LACHMI-001) individuals, collected before the CHMI (A) or at all timepoints together (B). Colour of the cells represent the Spearman’s rho value, ranging from -1 to +1. * p<0.05, ** p<0.01, *** p<0.001.
